# Pain and Efficacy Rating of a Microprocessor-Controlled Metered Injection System for Local Anaesthesia in Minor Hand Surgery

**DOI:** 10.1155/2011/362396

**Published:** 2011-05-25

**Authors:** André S. Nimigan, Bing Siang Gan

**Affiliations:** ^1^The Hand and Upper Limb Centre, St. Joseph's Health Centre, The University of Western Ontario, London, ON, Canada N6A 4L6; ^2^Division of Plastic Surgery, University of Western Ontario, London, Ontario, Canada N6A 4L6; ^3^Division of Orthopedic Surgery, University of Western Ontario, London, ON, Canada N6A 4L6; ^4^Department of Physiology and Pharmacology, University of Western Ontario, London, ON, Canada N6A 5C1; ^5^Department of Medical Biophysics, University of Western Ontario, London, ON, Canada N6A 5C1

## Abstract

*Purpose*. Little attention has been given to syringe design and local anaesthetic administration methods. A microprocessor-controlled anaesthetic delivery device has become available that may minimize discomfort during injection. The purpose of this study was to document the pain experience associated with the use of this system and to compare it with use of a conventional syringe. *Methods*. A prospective, randomized clinical trial was designed. 40 patients undergoing carpal tunnel release were block randomized according to sex into a two groups: a traditional syringe group and a microprocessor-controlled device group. The primary outcome measure was surgical pain and local anaesthetic administration pain. Secondary outcomes included volume of anaesthetic used and injection time. *Results*. Analysis showed that equivalent anaesthesia was achieved in the microprocessor-controlled group despite using a significantly lower volume of local anaesthetic (*P* = .0002). This same group, however, has significantly longer injection times (*P* < .0001). Pain during the injection process or during surgery was not different between the two groups. *Conclusions*. This RCT comparing traditional and microprocessor controlled methods of administering local anaesthetic showed similar levels of discomfort in both groups. While the microprocessor-controlled group used less volume, the total time for the administration was significantly greater.

## 1. Introduction

The use of local anaesthesia allows surgeons to perform minor surgery procedures in a variety of settings, including the emergency rooms and clinics. The first recorded nerve block was achieved by Halstead, who used cocaine to accomplish an inferior alveolar block on himself in 1884. Hollow tip hypodermic syringes were introduced not long after by Pravaz and Wood. Unfortunately, the administration of local anaesthesia in itself causes pain, despite attempts to diminish this anaesthesia-associated pain, such as by chemically modifying anaesthetic agents, adding buffering agents, or changing the anaesthetic temperature during administration. Very little attention has been given to the current syringe design and the administration methods, and effectively, syringe systems have changed a little since their introduction over a century ago [1]. 

A new development in the attempt to give greater operator control and minimize patient discomfort and distress is a product known as the Midwest Comfort Control System. This anaesthetic delivery device eliminates the variability of a thumb-operated plunger, allowing for maintenance of an ideal flow rate of anaesthetic [2]. The infusion rate is precisely regulated by a computer processor which immediately compensates for varying tissue resistance encountered in a single injection. In previous studies examining the effectiveness of pressure-regulated injection, it was found that when the flow rate and pressure of an injected anaesthetic were precisely controlled by a microprocessor, the injections were two to three times less painful than the manual injection (*P* < .001) [3]. Significant reductions in postoperative discomfort for an inferior alveolar nerve block have been demonstrated, and both users of the device as well as patients stated a preference for the microprocessor-controlled system [3]. The computerized anaesthesia delivery system has also been shown to provide significantly lower pain ratings for dental restorations [4] and reduce anxiety as well as pain and pain perception in the pediatric population [5, 6]. One study showed no difference in the pain behavior of children during the administration of local anaesthesia with a conventional injection or a computerized device when the operator was an experienced pediatric dentist [7]. The majority of these studies have come from the dental literature, but the device has also been studied and shown to be beneficial in minor anal surgery, toe surgery, and hair transplantation [8, 9]. The purpose of this study was to examine the benefits of this new injection system in minor hand surgery.

## 2. Methods

This single-centre, prospective randomized study was conducted at the Hand and Upper Limb Centre in London, Ontario, Canada. Approval was obtained from the institutional ethics review board prior to the beginning of the study. The objective of this study was to compare the pain, discomfort, and effectiveness of the traditional syringe method and a microprocessor-controlled delivery device for achieving local anaesthesia for carpal tunnel release surgery.

A permuted block design was used to randomize 40 adult patients undergoing open carpal tunnel release according to sex into two groups. One group was designated to receive local anaesthesia using traditional needle and syringe method, while patients in the second group received their anaesthesia using the microprocessor-controlled syringe system.

The initial sample size calculation was based on the highest standard deviation in reported VAS pain scores from a recent study looking at pain from open carpal tunnel release under local anaesthesia [10]. The alpha error in the study was set at 0.05, and the sample calculated to achieve a statistical power of 0.80 was 15.7 patients per group. 

The primary outcome measure was defined as the difference between the traditional needle and syringe group and the microprocessor-controlled system group measured by the validated visual analog scale (VAS) score for both perioperative surgical pain and pain related to the delivery of the local anaesthetic. This was accomplished with seven questions (Table 1).

Secondary outcomes of this study included the total volume of anaesthetic used, injection time, and the level of training of the practitioner administering the local anaesthetic. 

### 2.1. Recruitment

Consecutive subjects were recruited through the outpatient practice of the senior author. Potential subjects were assessed according to the inclusion and exclusion criteria (Table 4) in the study protocol and signed the informed consent prior to their procedure. 

### 2.2. Randomization

Sealed, opaque envelopes containing individual randomization assignments were prepared prior to the beginning of the study. Patients were sex matched, and the subjects' randomization was unveiled by the treating investigator just prior to the infiltration of the local anaesthetic. 

### 2.3. Treatment

All procedures were performed in the outpatient setting. The subjects were placed supine, and the appropriate arm was supported on an arm board. The site of injection was wiped with an alcohol swab, and the local anaesthetic was administered according to the randomization. The local anaesthetic used in both groups was 2% lidocaine without epinephrine, which was administered with either technique through a 30 gauge 1.0′ needle.

For the traditional group, a 10 cc syringe with a 30 gauge 1.0′ needle was used, and the operator administering the local anaesthetic was permitted to use the syringe in their own preferred method to administer local anesthesia. While the incision itself was standardized and always drawn by the senior author, the traditional syringe injection administration technique was deliberately not standardized. This was done in order to allow each individual physician to use the volume and approach with which they are most comfortable. For the purpose of this study, we considered this the “standard" of care, as opposed to the microprocessor-controlled group in which the injection volume was more uniform and the technique standardized in order to minimize the operator-dependent factors. It is worth emphasizing that there currently is no real “standard" for traditional injections, making the use of a microprocessor-controlled and standardized injection theoretically valuable in eliminating much of the user variability. 

For the group randomized to the microprocessor-controlled device, the Midwest Comfort Control System was used. Standard 1.8 cc cartridges containing 2% plain lidocaine were used and a 30 gauge 1.0′ needle was locked in position. The “block” setting on the programmable device was used for each injection. The needle was inserted, and local anaesthetic was infiltrated subcutaneously at the standard rate set by the microprocessor. Infiltration was carried out in the region of the standardized incision. All injections were performed by the senior author or plastic surgery residents. 

 Following the administration of local anaesthetic, a tourniquet was placed around the upper arm, and the subjects' hand and distal forearm were prepped using chlorhexidine solution and draped with sterile towels, and a standard open carpal tunnel release was then performed. Immediately following the procedure, patients were asked to fill out their VAS scores related to the seven VAS questions.

### 2.4. Statistical Analysis

Statistical analyses were performed using both one-sided *t*-tests and nonparametric Mann-Whitney tests, which make no assumption about the distribution of the data. The responses to VAS were analyzed both in 1 mm divisions (0–100) and in equal divisions of 1 cm (0–10). Subgroup analysis was also done looking at patients in whom the anaesthetic was delivered only by the senior consulting surgeon.

## 3. Results

Forty patients were randomized to either the traditional injection method (*n* = 20) or microprocessor-controlled (*n* = 20) groups. Due to the short-term nature of the intervention and immediate evaluation, no patients withdrew or were lost to followup. The two groups were found to be almost identical with respect to baseline demographics such as age, gender, and ethnicity. No adverse events (other than pain) were seen or reported during treatment or in the two-week followup. 

**Figure 1:**
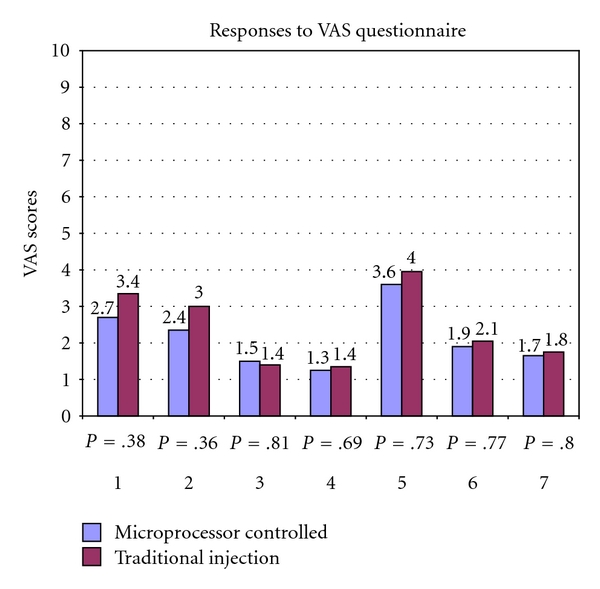
Responses to VAS pain questionnaire (see Table 1) and corresponding *P* values.

Using both parametric and nonparametric tests, the VAS data were analyzed both in the original 100 mm scale (Table 2) and in groups of ten divisions (one per centimeter, Table 3). With regard to primary outcome measure, no statistically significant differences were seen between the two groups' responses to all seven questions relating to pain during the injection and surgical procedure (Figure 1). Although the differences were not statistically significant, the microprocessor group showed numerically lower scores in response to all but one of the pain questions (how long did the pain last?).

When the analysis was limited to patients who were given local anaesthetic only by the senior consulting surgeon (no trainee involvement), again no statistically significant difference in pain responses was seen between the two groups.

**Figure 2:**
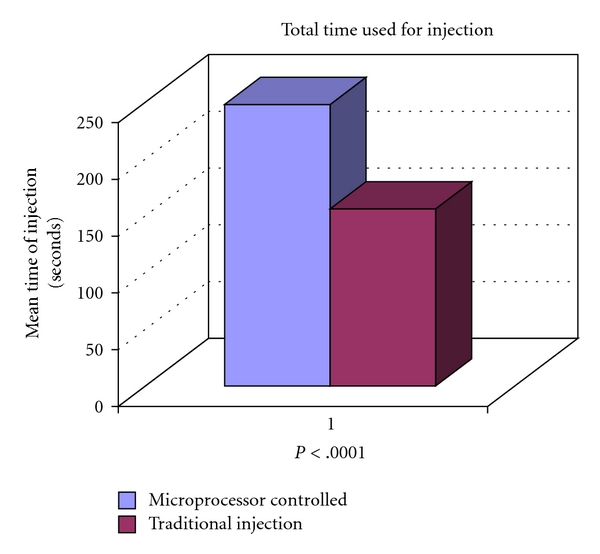
Total time required for infusion during microprocessor-controlled injection versus traditional injection (*P* < .0001).

**Figure 3:**
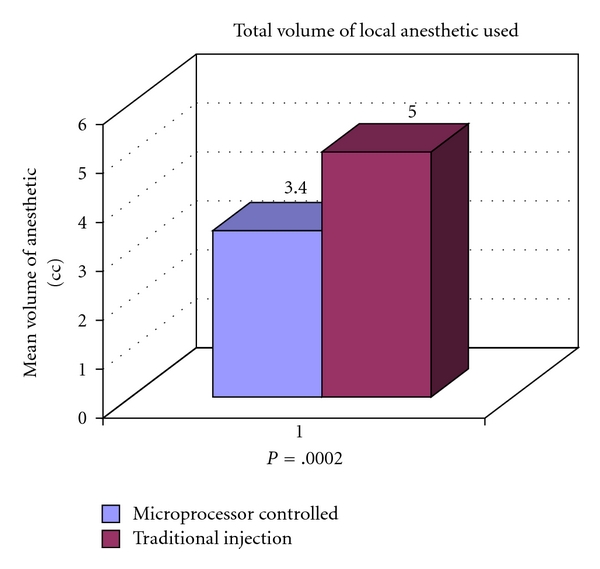
Total volume of local anaesthetic used during microprocessor-controlled injection versus traditional injection (*P* = .0002).

There was a significant difference between the two groups in both the time taken for the injection process (Figure 2) and the volume of local anaesthetic used for injection (Figure 3). The mean time taken for injection in the microprocessor-controlled group was longer, requiring 248 seconds compared to 156 seconds in the traditional group (*P* < .0001). On the other hand, the total volume of local anaesthetic used in the microprocessor-controlled group was less, with a mean of 3.4 cc being used compared to 5.0 cc in the traditional group (*P* = .0002). 

## 4. Discussion

The use of local anaesthesia allows surgeons to perform many procedures without the use of general anaesthesia in clinic and emergency room settings. It is well known that the administration of local anaesthetics is painful in and of itself. 

Three components are commonly identified as contributing to the pain experienced by the patient during injection. The first is the actual puncture of the skin with the hypodermic needle, and the second is the tissue distension caused by the injection of the solution. The painful burning sensation during injection comes primarily from administering the anaesthetic too rapidly or with too much force. Thirdly, the acidic pH of the commonly used amide local anaesthetics (for shelf-life preservation) causes local irritation which also promotes pain. Several approaches have been tried to reduce pain associated with the administration of local anaesthetics including penetration of the skin in a previously anesthetized area, slow injection, warming the infiltrate, and buffering the solution with sodium bicarbonate which are techniques traditionally used to minimize the discomfort experienced during infiltration of local anaesthetic agents. Interestingly, the traditional syringe design has not effectively changed since its original inception almost 150 years ago.

The traditional syringe design employs an awkward thumb-palm grasp that requires the user to place the needlepoint with precision while holding the syringe a certain distance from the point of insertion. The pump action that delivers the local anaesthetic requires forearm muscles that are far from the needle, making antagonist muscle activity almost impossible to avoid. In addition, conventional syringes do not allow precise control of the flow rate, and while slow injections are possible, the mechanics are challenging [11]. Injections into dense tissues such as the palate may require pressures up to 660 psi, possibly making control of a syringe even more difficult, erratic, and uncomfortable [2]. According to its manufacturers, the Midwest Comfort Control System anaesthetic delivery device (the “Wand”) eliminates the variability of a thumb-operated plunger, possibly allowing for maintenance of an ideal flow rate of anaesthetic [2]. The infusion rate is precisely regulated by a computer processor which immediately compensates for varying tissue resistance encountered in a single injection. The Wand's handpiece is a penlike plastic handle designed to give greater tactile feedback, precision, and operator ease, allowing for concentration on needle position and patient interaction, while patients find the penlike design less threatening [2, 3]. 

Needle deflection, a common cause of anaesthetic failure, is the result of the tissue forces on the beveled surface of the needle [12]. The use of a bidirectional rotational insertion technique of the needle with a beveled needle allows the user to overcome the perpendicular force vectors that would ordinarily lead to deflection of the needle shaft, irrespective of needle length, gauge, bevel design, or composition [13]. Bidirectional rotational insertion also seems to significantly reduce the force required to penetrate a substance, which is particularly relevant for those injections that penetrate dense connective tissues [14]. The Wand CCS claims to allow for maximization of this insertion technique due to its penlike grasp [3, 8, 9].

A large number of studies, mainly in the dental literature, have been published comparing the Comfort Control Syringe with traditional means of delivering local anaesthetic. This literature is overwhelmingly in favour of the Wand, with all but one study, that compared the pain behavior of children for both maxillary infiltration and mandibular blocks [7], indicating a significantly less painful patient experience with the microprocessor-ontrolled wand device. Analysis of our final data showed that equivalent anaesthesia was achieved in the microprocessor-controlled group despite using a significantly lower volume of local anesthetic. This same group, however, has significantly longer injection times. In contract to the existing literature, in the current study, none of the seven questions about pain during the injection process or during surgery showed any significant differences in visual analog scale reporting between the two groups. It was the impression of the operators that most patients were less anxious and exhibited less pain behavior such as grimacing or other signs of discomfort. The latter did not express itself in the hard numbers, and, in contrast to most of the previously published results, our randomized control trial comparing traditional and microprocessor-controlled methods of administering local anesthetic for carpal tunnel surgery showed no appreciable benefit in terms of reported levels of discomfort. It should be noted that this is the first study using the microprocessor-controlled syringe in hand surgery, and the first reported head-to-head trial using this device in adults that did not show improved patient comfort during the administration of local anesthesia. Our study did show that significantly lower anaesthetic volumes were required to achieve the same level of anaesthesia. 

Possible explanations for the failure of the Wand device to improve pain experience are that the Wand device was designed originally for use in dental procedures where tissue resistance, especially in palatal injections, can be quite high. Dental procedures and injections have developed a “reputation” for being painful, and patient anxiety around these procedures can be quite high. It is possible that injections into the palm and wrist for carpal tunnel surgery are less painful due to the inherently lower amount of tissue resistance and resultant tissue distension met during infiltration in this location. It can also be argued that it is technically easier to access the distal forearm for injections and less psychologically intimidating for patients when compared to intraoral injections. In addition, in our centre it is customary that local injections are given at a very slow rate, as evidenced by the relatively long 156 seconds that it takes on average to inject 5 cc of local anaesthetic using the conventional syringe.

These factors together may explain the minimally traumatic experience on the whole seen in this study for patients undergoing local anaesthetic infiltration for open carpal tunnel release surgery. In fact, in response to the question “How do you rate the discomfort of the freezing?” both groups had very low scores on the VAS for pain, with the microprocessor group and traditional group scoring 1.9 and 2.1, respectively, on a scale of zero to ten. Figure 1 demonstrates the minimal amount of pain reported by both groups, with no mean pain scores greater than four out of ten. In addition, the only question that leads to pain responses in either group that were close to four out of ten was regarding pain felt from the tourniquet during the procedure, and not from the injection process at all.

Perhaps then, with a comparatively painless injection to start with, the advantages of the microprocessor-controlled Wand device are simply not seen in blocks for minor hand surgery, and the advantages of this system may lie in procedures requiring injections of local anaesthetics into areas of high tissue resistance where the infiltration rate limiting effect of the microprocessor device is most tangible. This explanation, however, does not necessarily hold up in light of the few studies that have shown the microprocessor-controlled device to be highly beneficial in minor anal surgery, toe surgery, and hair transplantation [8, 9]. It is possible that the clinicians delivering the local anaesthetic in these studies did not habitually employ slow, gradual infiltration during the injections using the traditional syringe design, thereby highlighting the reduction in pain from tissue distension seen with the low, microprocessor-controlled flow rates of the Wand device. 

In conclusion, further studies using the Midwest Comfort Control System in hand surgery, particularly looking at procedures other than carpal tunnel release surgery, would be useful in determining whether this device has a place in hand surgery. 

## Figures and Tables

**Table 1:** Primary outcome measure questions asked to elicit scores on the visual analog scale (VAS) for patients undergoing carpal tunnel release.

Question 1	How much pain did you experience when the needle was inserted?
Question 2	How much pain did you experience during the injection of the freezing?
Question 3	How long did the pain last?
Question 4	How much pain did you feel during the surgery?
Question 5	How much pain did you feel from the tourniquet?
Question 6	How would you rate the discomfort of the freezing?
Question 7	How would you rate the discomfort of the surgery?

**Table 2:** Results for comparison of primary outcome measure (VAS) using single-sided *t*-test for visual analog scales 0–100 mm. See Table 1 for questions.

	Microprocessor-controlled injection	Traditional method of injection	*P* value
	*n* = 20 [Mean (SD)]	*n* = 20 [Mean (SD)]	
Question 1	22 (23)	29 (22)	.34
Question 2	18 (22)	26 (23)	.30
Question 3	8 (17)	9 (9)	.95
Question 4	4 (9)	6 (10)	.49
Question 5	29 (36)	34 (29)	.65
Question 6	13 (17)	15 (16)	.67
Question 7	11 (15)	12 (13)	.75
Volume of anesthetic used	3.4 (0.1)	5 (2)	.0002
Total injection time (seconds)	248 (39)	156 (54)	<.0001

**Table 3:** Results for comparison of primary outcome measures (VAS) using single-sided *t*-test for visual analog scales broken into ten category divisions. See Table 1 for questions.

	Microprocessor-controlled injection	Traditional method of injection	*P* value
	*n* = 20 [Mean (SD)]	*n* = 20 [Mean (SD)]	
Question 1	2.7 (2.3)	3.4 (2.3)	.38
Question 2	2.4 (2.2)	3.0 (2.3)	.36
Question 3	1.5 (1.6)	1.4 (0.8)	.81
Question 4	1.3 (0.7)	1.4 (0.8)	.69
Question 5	3.6 (3.5)	4.0 (2.9)	.73
Question 6	1.9 (1.7)	2.1 (1.5)	.77
Question 7	1.7 (1.3)	1.8 (1.2)	.80

**Table 4:** Study inclusion and exclusion criteria.

*Inclusion criteria*	
(i) Subject able to give informed consent	
(ii) Subject greater than 18 years old	
(iii) Predetermined need to undergo unilateral open carpal tunnel release	

*Exclusion criteria*	
(i) Subject unable to give informed consent	
(ii) Pregnant women	
(iii) Known sensitivity or allergy to Lidocaine	
(iv) Minors (age < 18)	
